
JOURNAL INFORMATION
==============================
NLM Title Abbreviation: ScientificWorldJournal
Journal ID: ScientificWorldJournal
NLM Journal ID: 101131163
Journal ID: TSWJ

The Scientific World Journal

ISSN: 2356-6140
EISSN: 1537-744X
Publisher: Wiley

ARTICLE INFORMATION
==============================
PMCID: PMC5917373
PMID: 16604254
DOI: 10.1100/tsw.2006.84
Article ID: 860581
Article version: 1
Subjects: Directions in Science

Microbial-induced Regulation of Syndecan Expression: Important Host Defense Mechanism or an Opportunity for Pathogens?

Smith Michael F. *
Gautam Jitendra K.
Black Steven G.
Ernst Peter B.
University of Virginia Health System, Departments of Medicine, Digestive Health Center of Excellence and Microbiology, Charlottesville, VA, USA
*Michael F. Smith: mfs3k@virginia.eduAcademic Editor: Martin Goette

Publication date: 2006
Electronic publication date: 2006 Apr 4
Volume: 6
First page: 442
Last page: 445
Received 2006 Feb 28; Accepted 2006 Mar 24
Copyright: Copyright © 2006 Michael F. Smith et al.
Copyright year: 2006
License: This is an open access article distributed under the Creative Commons Attribution License, which permits unrestricted use, distribution, and reproduction in any medium, provided the original work is properly cited.
License URL: https://creativecommons.org/licenses/by/3.0/

Keywords: syndecan, heparan sulfate proteoglycan, Toll-like receptor, TLR, Helicobacter, inflammation, NF-kappaB, NF-κB

Funding: National Institutes of Health RO1-AI34358 American Cancer Society RSG-01-034-TBE UVA Digestive Health Research Center DK56703

==============================
Directions in Science
*TheScientificWorldJOURNAL* (2006) 6, 442–445
ISSN 1537-744X; DOI 10.1100/tsw.2006.84

TheScientificWorldJOURNAL
www.thescientificworld.com

### Microbial-Induced Regulation of Syndecan Expression: Important Host Defense Mechanism or an Opportunity for Pathogens?

Michael F. Smith, Jr.*, Jitendra K. Gautam, Steven G. Black, and Peter B. Ernst

*University of Virginia Health System, Departments of Medicine, Digestive Health Center of Excellence and Microbiology, Charlottesville, VA*

**E-mail:** mfs3k@virginia.edu

*Received February 28, 2006; Accepted March 24, 2006; Published April 4, 2006*

---

**KEYWORDS:** syndecan; heparan sulfate proteoglycan; Toll-like receptor, TLR; *Helicobacter*; inflammation; NF-kappaB, NF-κB

---

Throughout evolution, microbes have developed a variety of means for subverting the host's normal cellular components in order to aid in their colonization of host tissue. Similarly, the host has devised a plethora of responses aimed at preventing colonization or at least ridding itself of the infectious agent once it has gained a foothold. Often times, determining whether a response is more beneficial to the host or to the invading pathogen can be difficult.

The syndecans are a family of four, type-I transmembrane heparan sulfate proteoglycans (HSPGs) that, together with the lipid-linked glypicans, represent the major source of heparan sulfate on the cell surface. The syndecans bind to a wide variety of soluble and insoluble extracellular effector molecules such as extracellular matrix components, growth factors, cytokines, and microbial pathogens. In a study recently published in *Glycobiology*, we examined the expression of syndecan-4 in gastric epithelial cells and macrophages[1]. In response to infection with the human gastric pathogen *Helicobacter pylori*, gastric epithelial cells increased the expression of syndecan-4 as measured both by mRNA levels and cell surface staining. Furthermore, we demonstrated that syndecan-4 expression could also be increased by stimulation of the epithelial cells or macrophages with agonists specific for members of the Toll-like receptor (TLR) family that are the principal cell surface receptors responsible for sensing microbial associated products. Like a great many other genes involved in the inflammatory response, enhanced gene expression was shown to be dependent on binding of the NF-κB transcription factor to a specific sequence within the promoter of the human syndecan-4 gene. Our findings thus add to the growing body of evidence that suggests that syndecan-4 may play a significant role in the host response to microbial infection.

Several previous studies have indicated that syndecan-4 expression can be enhanced during an inflammatory response. Two *in vitro* studies indicated that syndecan-4 expression could be enhanced in endothelial cells or HeLa cells in response to TNF-α, a cytokine commonly induced during inflammation and infection[2,3]. Furthermore, a 2001 study from Ishiguro and colleagues demonstrated increased syndecan-4 expression in the liver and macrophages in a mouse endotoxemia model[4]. Perhaps more intriguingly, that same study demonstrated increased endotoxin-induced mortality in syndecan-4–

*Corresponding author.
©2006 with author.
Published by TheScientificWorld, Ltd.; www.thescientificworld.com deficient mice perhaps as a result of a decreased ability of the macrophages to bind TGF-β, which acts to inhibit inflammatory responses. Syndecan-2 has also been indicated to be expressed inducibly in human monocyte-derived macrophages[5]. Conversely, syndecan-1 expression in two colonic epithelial cell lines is down-regulated by treatment with TNF-α or IL-1β[6]. Thus, ample evidence is now accumulating that shows that levels of syndecan expression can be regulated during an inflammatory response. While this response to injury or infection may be viewed by the host as beneficial during the healing and resolution phase via enhancing responses to cytokines, growth factors, and chemokines (examples of which have been variably shown to bind syndecan family members[7,8,9]), it also appears that pathogens too have evolved the means to use HSPGs to their own benefit.

Many pathogens have been shown to bind specifically to HSPGs expressed on eukaryotic cell surfaces[10]. Furthermore, an increasing number of published studies are demonstrating links between the syndecan family members, in particular syndecans-1 and -4, and bacterial or viral infections. Roles for syndecan-1 and -4 in mediating bacterial/epithelial cell interactions were first demonstrated in a paper by Freissler et al.[11]. When overexpressed in HeLa cells, both syndecan-1 and -4 led to increased *Neisseria gonnorrhoeae* invasion, which was critically dependent on the presence of the cytoplasmic domains of both syndecans. These results imply that the syndecans are not only serving as receptors for the bacteria, but in fact, play an active role in the invasive process by transmitting a signal to the cytoskeleton. Other human pathogenic bacteria including *Orientia tsutsugamushi* (the causative agent of scrub typhi)[12], *Listeria monocytogenes, Streptococcus pyogenes,* and *Staphylococcal* sp., among others[13], have also been demonstrated to utilize syndecans-1 and/or -4 for binding to cell surfaces. Like a number of other cell surface receptors, the syndecan ectodomains are shed from the cell surface both constitutively during the course of heparan sulfate turnover and in a regulated fashion in response to tissue injury, growth factor stimulation, and microbial infection[14,15]. At least one pathogen, *Pseudomonnas aeroginosa*, has learned to use shed syndecan-1 for its own benefit[16,17,18]. *P. aeruginosa*, through its virulence factor LasA, induces the *in vitro* shedding of syndecan-1 from epithelial cells[17]. Surprisingly, syndecan-1–deficient mice are more resistant to *P. aeruginosa* infection, but become susceptible when given purified syndecan-1 ectodomains, indicating that *P. aeruginosa* exploits the shedding of syndecan-1 ectodomains as a virulence factor[16]. Syndecan-1 ectodomain shedding has also been observed to be induced by the α and β toxins of *Staphyloccous aureus*, suggesting that this may be a common virulence mechanism used by a variety of bacterial species.

While we have demonstrated that syndecan-4 expression can be significantly up-regulated in response to *H. pylori* infection, the implications of this response remain unclear. Like other pathogens discussed above, *H. pylori* may exploit cell surface HSPGs in order to bind host cells[20,21,22]. Ruiz-Bustos and colleagues identified several heparan sulfate binding proteins (HSBPs) from *H. pylori* that could potentially interact with soluble or cell surface HSPGs[23]. The same group also demonstrated that a 71.5-kDa extracellular HSBP could, in fact, participate in the binding of *H. pylori* to either HeLa S3 or Kato III cells[24]. Additionally, Utt et al. have provided evidence suggesting that the vaculating toxin of *H. pylori*, *vacA*, may utilize heparan sulfate as a cell surface receptor[20]. Thus, there is significant evidence to suggest that HSPGs expressed on the surface of epithelial cells may participate in the host response to *H. pylori* infection. How the syndecans participate in the host response to *H. pylori* remains open to speculation. As illustrated in Fig. 1, one mechanism would suggest that *H. pylori* utilizes syndecans to bind to the gastric epithelial surface as an aid in colonization. However, a second possible model can be envisioned whereby the shedding of syndecan ectodomains from the cell surface plays a protective role by acting as a soluble "decoy receptor" thereby decreasing *H. pylori* binding to the cell. These issues are currently being addressed in our laboratory and it is hoped that the results of these studies will open up new avenues for the use of syndecan-based reagents as therapeutics for the treatment of *H. pylori* and other microbial infections.

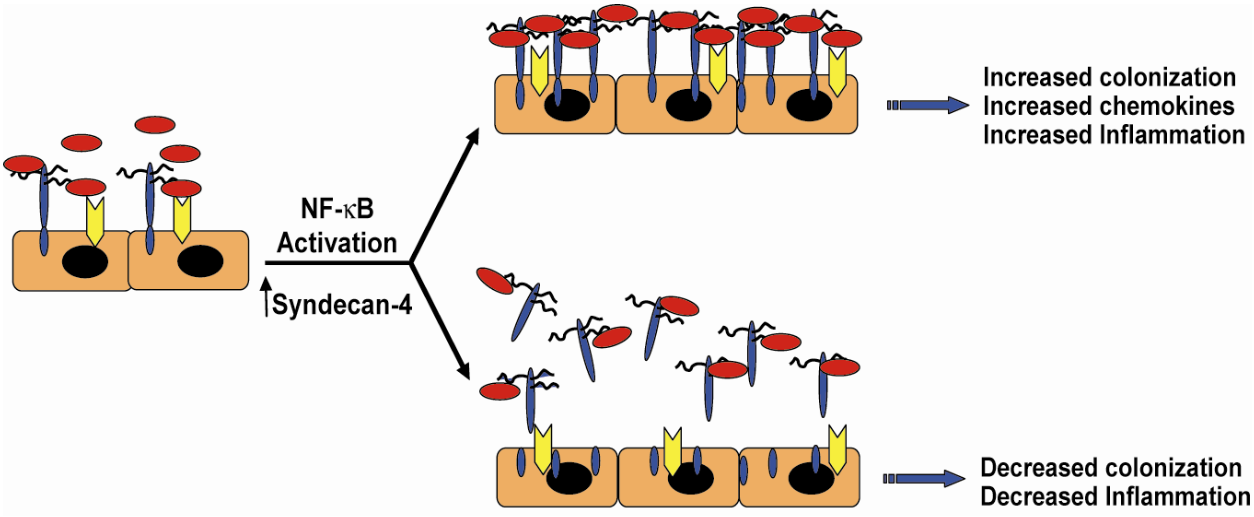

**FIGURE 1.** Possible effects of increased syndecan-4 expression on microbe/epithelial interactions. *H. pylori* (red) binds to syndecan-4 (blue) on the surface of epithelial cells resulting in the activation of TLR (yellow). TLR ligation activates NF-κB and induces the expression of syndecan-4. In one model, the increased expression of syndecan-4 on the surface results in increases in binding of the bacteria, inflammation, and subsequent tissue damage. Alternatively, syndecan-4 shed from the surface of activated epithelial cells acts as a "decoy receptor" for the bacteria resulting in decreases in both colonization and inflammation thereby acting as a protective mechanism.

### ACKNOWLEDGMENTS

This work was support by grants to M.F.S. from the National Institutes of Health (RO1-AI34358) and the American Cancer Society (RSG-01-034-TBE). Support from the Immunology and Cell Isolation Core of the UVA Digestive Health Research Center (DK56703) is also gratefully acknowledged.

### REFERENCES

1. Smith, M.F., Jr., Novotny, J., Carl, V.S., and Comeau, L.D. (2006) Helicobacter pylori and toll-like receptor agonists induce syndecan-4 expression in an NF-{kappa}B-dependent manner. *Glycobiology* **16**, 221.
2. Zhang, Y., Pasparakis, M., Kollias, G., and Simons, M. (1999) Myocyte-dependent regulation of endothelial cell syndecan-4 expression. Role of TNF-alpha. *J. Biol. Chem.* **274**, 14786.
3. Zhou, A., Scoggin, S., Gaynor, R.B., and Williams, N.S. (2003) Identification of NF-kappa B-regulated genes induced by TNFalpha utilizing expression profiling and RNA interference. *Oncogene* **22**, 2054.
4. Ishiguro, K., Kadomatsu, K., Kojima, T., Muramatsu, H., Iwase, M., Yoshikai, Y., Yanada, M., Yamamoto, K., Matsushita, T., Nishimura, M., Kusugami, K., Saito, H., and Muramatsu, T. (2001) Syndecan-4 deficiency leads to high mortality of lipopolysaccharide-injected mice. *J. Biol. Chem.* **276**, 47483.
5. Clasper, S., Vekemans, S., Fiore, M., Plebanski, M., Wordsworth, P., David, G., and Jackson, D.G. (1999) Inducible expression of the cell surface heparan sulfate proteoglycan syndecan-2 (fibroglycan) on human activated macrophages can regulate fibroblast growth factor action. *J. Biol. Chem.* **274**, 24113.
6. Day, R.M., Mitchell, T.J., Knight, S.C., and Forbes, A. (2003) Regulation of epithelial syndecan-1 expression by inflammatory cytokines. *Cytokine* **21**, 224.
7. Gotte, M. (2003) Syndecans in inflammation. *FASEB J.* **17,** 575.
8. Li, Q., Park, P.W., Wilson, C.L., and Parks, W.C. (2002) Matrilysin shedding of syndecan-1 regulates chemokine mobilization and transepithelial efflux of neutrophils in acute lung Injury. *Cell* **111**, 635.
9. Marshall, L.J., Ramdin, L.S., Brooks, T., DPhil, P.C., and Shute, J.K. (2003) Plasminogen activator inhibitor-1 supports IL-8-mediated neutrophil transendothelial migration by inhibition of the constitutive shedding of endothelial IL-8/heparan sulfate/syndecan-1 complexes. *J. Immunol.* **171**, 2057.
10. Wadstrom, T. and Ljungh, A. (1999) Glycosaminoglycan-binding microbial proteins in tissue adhesion and invasion: key events in microbial pathogenicity. *J. Med. Microbiol.* **48,** 223.
11. Freissler, E., Meyer auf der Heyde, A., David, G., Meyer, T.F., and Dehio, C. (2000) Syndecan-1 and syndecan-4 can mediate the invasion of OpaHSPG-expressing Neisseria gonorrhoeae into epithelial cells. *Cell. Microbiol.* **2**, 69.
12. Kim, H.R., Choi, M.S., and Kim, I.S. (2004) Role of syndecan-4 in the cellular invasion of Orientia tsutsugamushi.

*Microb. Pathog.* **36,** 219.

13. Henry-Stanley, M.J., Hess, D.J., Erickson, E.A., Garni, R.M., and Wells, C.L. (2003) Role of heparan sulfate in interactions of Listeria monocytogenes with enterocytes. *Med. Microbiol. Immunol (Berl.)* **192**, 107.
14. Fitzgerald, M.L., Wang, Z., Park, P.W., Murphy, G., and Bernfield, M. (2000) Shedding of syndecan-1 and -4 ectodomains is regulated by multiple signaling pathways and mediated by a TIMP-3-sensitive metalloproteinase. *J. Cell Biol.* **148**, 811.
15. Subramanian, S.V., Fitzgerald, M.L., and Bernfield, M. (1997) Regulated shedding of syndecan-1 and -4 ectodomains by thrombin and growth factor receptor activation. *J. Biol. Chem.* **272,** 14713.
16. Park, P.W., Pier, G.B., Hinkes, M.T., and Bernfield, M. (2001) Exploitation of syndecan-1 shedding by Pseudomonas aeruginosa enhances virulence. *Nature* **411**, 98.
17. Park, P.W., Pier, G.B., Preston, M.J., Goldberger, O., Fitzgerald, M.L., and Bernfield, M. (2000) Syndecan-1 shedding is enhanced by LasA, a secreted virulence factor of Pseudomonas aeruginosa. *J. Biol. Chem.* **275**, 3057.
18. Haynes, A., III, Ruda, F., Oliver, J., Hamood, A.N., Griswold, J.A., Park, P.W., and Rumbaugh, K.P. (2005) Syndecan 1 shedding contributes to Pseudomonas aeruginosa sepsis. *Infect. Immun.* **73**, 7914.
19. Park, P.W., Foster, T.J., Nishi, E., Duncan, S.J., Klagsbrun, M., and Chen, Y., 2004. Activation of syndecan-1 ectodomain shedding by Staphylococcus aureus alpha-toxin and beta-toxin. *J Biol Chem.* **279**, 251.
20. Utt, M., Danielsson, B., and Wadstrom, T. (2001) Helicobacter pylori vacuolating cytotoxin binding to a putative cell surface receptor, heparan sulfate, studied by surface plasmon resonance. *FEMS Immunol. Med. Microbiol.* **30**, 109.
21. Utt, M. and Wadstrom, T. (1997) Identification of heparan sulphate binding surface proteins of Helicobacter pylori: inhibition of heparan sulphate binding with sulphated carbohydrate polymers. *J. Med. Microbiol.* **46**, 541.
22. Duensing, T.D., Wing, J.S., and van Putten, J.P. (1999) Sulfated polysaccharide-directed recruitment of mammalian host proteins: a novel strategy in microbial pathogenesis. *Infect. Immun.* **67,** 4463.
23. Ruiz-Bustos, E., Ochoa, J.L., Wadstrom, T., and Ascencio, F. (2001) Isolation and characterisation of putative adhesins from Helicobacter pylori with affinity for heparan sulphate proteoglycan. *J. Med. Microbiol.* **50**, 215.

24 Guzman-Murillo, M.A., Ruiz-Bustos, E.D.U.A., Ho, B.O.W., and Ascencio, F.E.L.I. (2001) Involvement of the heparan sulphate-binding proteins of Helicobacter pylori in its adherence to HeLa S3 and Kato III cell lines. *J. Med. Microbiol.* **50**, 320.

**This article should be cited as follows:**

Smith, M.F., Jr., Gautam, J.K., Black, S.G., and Ernst, P.B. (2006) Microbial-induced regulation of syndecan expression: important host defense mechanism or an opportunity for pathogens? *TheScientificWorldJOURNAL* **6**, 442–445. DOI 10.1100/tsw.2006.84.

### BIOSKETCH

**Michael F. Smith, Jr.** obtained his Ph.D. in Microbiology and Immunology from Temple University (Philadelphia, PA) in 1989. From 1989–1996, he studied the regulation, expression, and function of the interleukin-1 receptor antagonist (IL-1Ra) in the laboratory of Dr. William Arend in the Division of Rheumatology at the University of Colorado School of Medicine (Denver). In 1996, he moved to the Division of Gastroenterology at the University of Virginia (Charlottesville) as an Assistant Professor, where he continued to study the transcriptional regulation of IL-1Ra in macrophages stimulated through Toll-like receptors. In 2002, he was appointed Associate Professor of Medicine and Microbiology in the Digestive Health Center of Excellence at the University of Virginia. His laboratory continues to study how microbial infections or Toll-like receptor agonists influence responses in epithelial cells and macrophages.
